# Expression profiling of cerebrospinal fluid identifies dysregulated antiviral mechanisms in multiple sclerosis

**DOI:** 10.1093/brain/awad404

**Published:** 2023-12-01

**Authors:** Maria Ban, Danila Bredikhin, Yuanhua Huang, Marc Jan Bonder, Kania Katarzyna, Amanda J Oliver, Nicola K Wilson, Paul Coupland, James Hadfield, Berthold Göttgens, Elo Madissoon, Oliver Stegle, Stephen Sawcer

**Affiliations:** Department of Clinical Neurosciences, University of Cambridge, Cambridge CB2 0QQ, UK; European Molecular Biology Laboratory, Genome Biology Unit, 69117 Heidelberg, Germany; Division of Computational Genomics and Systems Genetics, German Cancer Research Center (DKFZ), 69120 Heidelberg, Germany; Department of Clinical Neurosciences, University of Cambridge, Cambridge CB2 0QQ, UK; European Molecular Biology Laboratory, European Bioinformatics Institute, Cambridge CB10 1SD, UK; European Molecular Biology Laboratory, Genome Biology Unit, 69117 Heidelberg, Germany; Division of Computational Genomics and Systems Genetics, German Cancer Research Center (DKFZ), 69120 Heidelberg, Germany; University of Cambridge, CRUK Cambridge Institute, Cambridge CB2 0RE, UK; Wellcome Sanger Institute, Wellcome Genome Campus, Cambridge CB10 1SA, UK; Department of Haematology, University of Cambridge, Cambridge CB2 0AW, UK; Wellcome-Medical Research Council Cambridge Stem Cell Institute, University of Cambridge, Cambridge CB2 0AW, UK; University of Cambridge, CRUK Cambridge Institute, Cambridge CB2 0RE, UK; University of Cambridge, CRUK Cambridge Institute, Cambridge CB2 0RE, UK; Department of Haematology, University of Cambridge, Cambridge CB2 0AW, UK; Wellcome-Medical Research Council Cambridge Stem Cell Institute, University of Cambridge, Cambridge CB2 0AW, UK; European Molecular Biology Laboratory, European Bioinformatics Institute, Cambridge CB10 1SD, UK; Wellcome Sanger Institute, Wellcome Genome Campus, Cambridge CB10 1SA, UK; European Molecular Biology Laboratory, Genome Biology Unit, 69117 Heidelberg, Germany; Division of Computational Genomics and Systems Genetics, German Cancer Research Center (DKFZ), 69120 Heidelberg, Germany; European Molecular Biology Laboratory, European Bioinformatics Institute, Cambridge CB10 1SD, UK; Department of Clinical Neurosciences, University of Cambridge, Cambridge CB2 0QQ, UK

**Keywords:** multiple sclerosis, scRNA-seq, eQTL, CSF, ZC3HAV1, IFITM2

## Abstract

Despite the overwhelming evidence that multiple sclerosis is an autoimmune disease, relatively little is known about the precise nature of the immune dysregulation underlying the development of the disease.

Reasoning that the CSF from patients might be enriched for cells relevant in pathogenesis, we have completed a high-resolution single-cell analysis of 96 732 CSF cells collected from 33 patients with multiple sclerosis (*n* = 48 675) and 48 patients with other neurological diseases (*n* = 48 057).

Completing comprehensive cell type annotation, we identified a rare population of CD8+ T cells, characterized by the upregulation of inhibitory receptors, increased in patients with multiple sclerosis. Applying a Multi-Omics Factor Analysis to these single-cell data further revealed that activity in pathways responsible for controlling inflammatory and type 1 interferon responses are altered in multiple sclerosis in both T cells and myeloid cells. We also undertook a systematic search for expression quantitative trait loci in the CSF cells. Of particular interest were two expression quantitative trait loci in CD8+ T cells that were fine mapped to multiple sclerosis susceptibility variants in the viral control genes *ZC3HAV1* (rs10271373) and *IFITM2* (rs1059091). Further analysis suggests that these associations likely reflect genetic effects on RNA splicing and cell-type specific gene expression respectively.

Collectively, our study suggests that alterations in viral control mechanisms might be important in the development of multiple sclerosis.

## Teaser

Ban *et al.* use single-cell RNA sequencing to explore the characteristics of cells within the CSF of patients with multiple sclerosis and other neurological diseases and find evidence that dysregulation of viral control mechanisms contributes to the development of multiple sclerosis.

See Schwab (https://doi.org/10.1093/brain/awae006) for a scientific commentary on this article.

## Introduction

Multiple sclerosis is a chronic autoimmune disease of the CNS which is pathologically characterized by patchy inflammatory demyelination and progressive neurodegeneration. Genome-wide association studies (GWAS) have identified over 200 susceptibility variants, the majority of which map to regulatory regions markedly enriched for genes with immunological functions,^1^ collectively suggesting a role for dysregulation in immune cell pathways in disease susceptibility. To date though, the analysis of circulating peripheral immune cells has provided limited insights into the aetiology of multiple sclerosis. Given the immune privilege of the CNS, we reasoned that the immune cells in the CSF are likely to be enriched for cells of relevance to multiple sclerosis and might therefore provide novel insights that might otherwise be obscured by non-multiple sclerosis relevant parts of the circulating immune system.

Abnormalities in the CSF, including an elevated lymphocyte count and the presence of oligoclonal immunoglobulin bands indicative of chronic immune activation, are hallmark features of the disease and are used to aid diagnosis.^2^ Immunophenotyping of CSF cells using flow cytometry has shown that patients with multiple sclerosis have increased proportions of B and plasma cells and decreased proportions of monocytes compared to healthy individuals or individuals with other neurological diseases.^3,4^ Furthermore, it has also been shown that cells within the CSF of multiple sclerosis patients are predominantly of a memory phenotype and have a more activated profile.^4^ Single-cell RNA sequencing (scRNA-seq) studies have corroborated these early findings and also refined specific CD4+ T cell subsets that have increased frequencies in patients with multiple sclerosis including T regulatory cells and T follicular helper cells, and have further proposed novel cell subtypes as potential key players in multiple sclerosis pathogenesis which require further investigation.^5-8^ However, these studies have lacked the power to explore the effects of known multiple sclerosis susceptibility variants in the CSF.

Here we use scRNA-seq to provide a high-resolution examination of CSF cell expression in 81 samples collected from patients with multiple sclerosis (*n* = 33) and from patients with other neurological conditions (*n* = 48). Following cell type annotation, we identified several cell-type-specific regulatory programs that correlated with disease status. Furthermore, we also undertook the first CSF-based single-cell expression quantitative trait loci (eQTL) analysis, which identified eQTL effects for two multiple sclerosis susceptibility variants in CD8+ T cells, the risk allele rs10271373_A increasing expression of the zinc finger CCCH-type antiviral protein 1 (*ZC3HAV1*) and rs1059091_A reducing the expression of interferon-induced transmembrane protein 2 (*IFITM2*). Overall, our observations suggest a possible dysregulation in the control of viral responses in multiple sclerosis patients.

## Materials and methods

### Patient recruitment

Our study was approved by the National Research Ethics Committee (Service South Central—Berkshire; 15/SC/0087) and all study subjects gave valid fully informed written consent. In total, 88 subjects were recruited from the Cambridge University Hospital (Addenbrooke’s) neurosciences department. Patients were recruited from amongst those attending the department for a lumbar puncture as part of the investigation and treatment of their neurological condition. For seven of the recruited patients (three multiple sclerosis; two ideopathic intracranial hypertension; one non-inflammatory neurological disease; and one other inflammatory neurological disease), the CSF sample was unusable for technical reasons. Multiple sclerosis was diagnosed in line with standard clinical criteria.^9^ All but two of our multiple sclerosis patients were newly diagnosed and therefore not on disease modifying treatment at the time of sampling. The two multiple sclerosis patients with an established diagnosis were both on treatment with natalizumab (Tysabri®). Disease activity in multiple sclerosis was determined by the primary clinical team on the basis of clinical and radiological features; the disease being judged to be inactive, active or highly active. Supplementary Table 1 shows the demographics, clinical and laboratory hospital measured CSF features for the 81 study subjects with RNA sequencing data that passed quality control and were included in the analysis. In the UK, lumbar puncture is not routinely performed in the investigation of suspected multiple sclerosis and therefore tends to be employed in the context of less typical cases. This selection of subjects for the investigation likely explains the older than average age at diagnosis of our multiple sclerosis cases, and the absence of the usual excess of females. The other inflammatory neurological diseases (OIND) include chronic inflammatory demyelinating polyneuropathy, neurosarcoidosis and transverse myelitis, while the non-inflammatory neurological diseases (NIND) include cerebrovascular disease, functional neurological disorder, low-grade glioma, migraine, myelopathy, neuropathy, seizure, trigeminal neuralgia and ideopathic intracranial hypertension (IIH)—the later were considered as a separate group as their CSF would not be expected to differ from normality other than in terms of pressure.

### Cell isolation and single cell library generation

All samples reached the lab within 1 hour of collection and were either processed immediately or frozen for later pooling. To each sample an equivalent volume of X-VIVO^TM^ 10 Serum-free Hematopoietic Cell Medium (Lonza) was added to maintain cell viability. Owing to the low concentration of cells in the CSF, each sample was first concentrated by centrifugation at 300*g* for 10 min, the supernatant removed leaving approximately a 200 μl volume. A manual cell count was completed on the concentrated sample using a Neubauer haemocytometer and cell viability assessed using Trypan Blue exclusion dye. The cell suspension was then centrifuged at 300*g* for another 10 min to further concentrate the sample to a final volume of 32 μl ready to be loaded onto a 10x Chromium Single Cell Controller using the Chromium Single Cell Gene Expression 3′ v2 kit (10x Genomics). Sequencing was completed on an Illumina HiSeq 4000 at the Cancer Research Institute, Cambridge University sequencing hub using locally optimized protocols and settings.

### Genotype imputation

All 81 donors were genotyped using the Illumina Global Screening Array. Imputation was performed against the Haplotype Reference Consortium (HRC)^10^ data using EAGLE2 through the Sanger Imputation server (https://imputation.sanger.ac.uk). Genotypes were converted to hard call binary plink format using GenotypeHarmonizer,^11^ before performing eQTL mapping. During the conversion we removed genotype calls with a posterior probability below 0.4, single nucleotide polymorphisms with a MACH R^2^ < 0.6 and/or a Hardy–Weinberg equilibrium below 1.0 ×10^−4^.

### Sequencing data preprocessing

The raw scRNA-seq reads in FASTQ format from each sequencing run were processed using Cell Ranger v3.1 (10x Genomics) and aligned to the human genome (Homo_sapiens.GRCh38.dna.primary_assembly.fa) using the default Cell Ranger settings and annotated using Homo_sapiens.GRCh38.93.filtered.gtf. Cells were called using both Cell Ranger and emptyDrops,^12^ with the union of cells called used for all downstream analysis. Among the 71 sequencing runs, five of these sequencing runs were multiplexed with two to four donors per run, in total 15 donors were included in multiplexed runs. To demultiplex these runs, we first used cellSNP v0.1.7^13^ to genotype each cell, followed by Vireo v0.2.1^14^ to assign cells to each multiplexed individual based on known genotypes. We used two separate strategies to detect doublets. First, for each multiplexed sequencing run, Vireo^14^ identifies the cross-donor doublets through their mixed genotypes. Second, we also detected cross-cell type doublets from the transcriptome profiles on each sequencing run by a consensus method combining doubletFinder^15^ and Scrublet.^16^

### Cell type annotation

The raw count matrix was corrected for ambient RNA using the soupX tool^17^ in R using standard settings. Count matrix normalization and log-transformation, clustering and cell type annotation were performed using the package scanpy^18^ in Python. Batch effects were reduced by using the BBKNN tool.^19^ Clusters were annotated to cell types according to known canonical marker genes. A sequential clustering method was then used for finer annotation of larger clusters. Clusters where there were markers from multiple lineages indicating contamination, markers of dividing cells, low unique molecular identifier (UMI) counts or high mitochondrial percentage were all removed prior to downstream analysis. Cell types were annotated to the finest achievable level. Related clusters were later combined into a higher level annotation that we used for downstream analysis that required a larger number of cells for each analysis. Differential cell type composition between groups was analysed using the DCATS R package (v0.99.7).

### Factor decomposition using multi-omics factor analysis

Multi-omics factor analysis (MOFA+)^20^ was applied to the normalized counts of highly-variable genes provided with cell type labels and set to learn 30 factors. Gene enrichment analysis was performed as implemented in the MOFA2 R package using the C5 ontology gene sets from the MSIGDB database.^21,22^ Factor weights and the DoRothEA database of transcription factor targets^23^ were then used to identify transcription factor regulons enriched in each factor. Factor weights for transcription factor targets were aggregated per factor and per transcription factor, which can be represented as a matrix product of factor weights and binary transcription factor—gene target relations. In order to test for the significance of the associations, for each MOFA+ factor and transcription factor, the same number of targets was sampled randomly 10 000 times in order to calculate an empirical *P*-value.

### Viral genome alignment

In this study, we performed a cell-resolution viral genome alignment. First, we compiled a list of 833 viral sequences, containing 762 viruses collected from VirTect,^24^ and seven other viruses and 64 consensus sequences of human endogenous retroviruses (HERVs) collected by Vargiu *et al*.^25^ Then for each of our scRNA-seq batches, we extracted the reads that were uncounted or unmapped for the human transcriptome in the CellRanger step by using ‘samtools view possorted_genome_bam.bam | grep -v ‘xf:i:25’>xf_non25.sam’. Next, we aligned these reads to the 833 viral sequences via STARsolo^26^ using CellRanger’s bam file as input and assigned each read to a specific cell using the cell-associated barcode list for each sample (see the ‘Sequencing data preprocessing’ section) as a whitelist. A cell-by-virus UMI count matrix is returned and by concatenating all batches we can perform downstream analysis. For reproducibility and public reuse, we have packaged all these analysis steps at https://github.com/huangyh09/ViralScan. To compare the donor-level prevalence between multiple sclerosis and non-multiple sclerosis samples, we performed a logistic regression to test if the coefficient of ‘is_MS’ is significantly non-zero with a model: Virus_present ∼ is_MS+total_UMIs+intercept. To compare the cell-level prevalence between multiple sclerosis and non-multiple sclerosis samples, we performed a linear regression to test if the coefficient of ‘is_MS’ is significantly non-zero with a model: viral_proportion ∼ is_MS+total_UMIs_log+total_cells_log+intercept.

### Expression quantitative trait loci mapping

For eQTL mapping, we followed the best practices for single-cell eQTL mapping outlined by Cuomo *et al*.^27^ Specifically, we did single cell-based normalization using sctransform^28^ and afterwards mean aggregated the cells to donor level gene-expression. The eQTLs were mapped per cell type using a linear mixed model implemented in LIMIX v2 (https://github.com/limix/limix). As outlined in Cuomo *et al*.,^27^ we leveraged two random effects, one to account for population structure and one for donor wise read-depth (encoded as 1/*n* reads per donor). In the analysis of each cell type, donors with fewer than two cells of that type were dropped, making the number of donors, and therefore power, different per cell type. Owing to the limited sample size of our study, we chose to only map eQTLs for protein coding genes that had non-zero expression in at least 10% of the donors and chose to filter SNPs on a minor allele frequency of 10% and used a cis-eQTL window of 100 kb either side of the gene.

Multiple testing correction was performed as in Cuomo *et al*.^27^ Specifically, we used 1000 permutations per QTL, and first correct per gene for testing multiple variants per gene followed by two different approaches to correct for the number of genes per eQTL map. For genes included in the eQTLGen^29^ set, we applied the conditional false discovery rate (cFDR) approach (conditioning on the eQTLGen summary statistics); for the remaining genes, we used the Storey Q value approach. FDR was controlled at FDR 10% and cFDR 10%. Effects were considered to be replicated in other tissues when the direction of effect was concordant and there was nominal statistical significance in the second tissue.

For fine-mapping, we applied SusieR v0.11.92^30^ using the default settings in each cell type separately following the guidelines provided by the authors. We annotated the eQTL signals based on fine-mapping results and specifically annotated signals overlapping with multiple sclerosis GWAS variants.

### Detection of allele specific expression

To perform cell type-specific allele specific expression (ASE), we first genotyped each individual cell with cellsnp-lite v1.2.0^13^ and aggregated expression data for each cell type to generate pseudo-bulk level data. We focused on the 8046 SNPs across 512 *cis*-genes of multiple sclerosis disease variants. Where there were multiple coding SNPs in a gene, we only considered the one with the maximum number of donors with both heterozygous genotypes and scRNA-seq data for that gene. Then for each SNP in each cell type, we use all informative donors to perform a beta-binomial regression with AOD R package (v1.3.1) and test if the mean allele frequency is 0.5, equivalent to testing if the intercept of the regression is 0. The *P*-value calculated with the Wald test is obtained.

### Analysis of splicing and transcript usage

We first simplified the transcript annotation of the gene *ZC3HAV1* into two major transcripts with distinct exons in the 3′ end. Then using BRIE v2.2.0^31^ we quantified the transcript compatible counts for each individual cell with brie-count using default parameters. A beta-binomial regression (from AOD R package) was used to calculate the *P*-value to test if there is a genetic effect on the proportion of transcript usage.

## Results

### The cellular architecture of CSF

**Figure 1:**
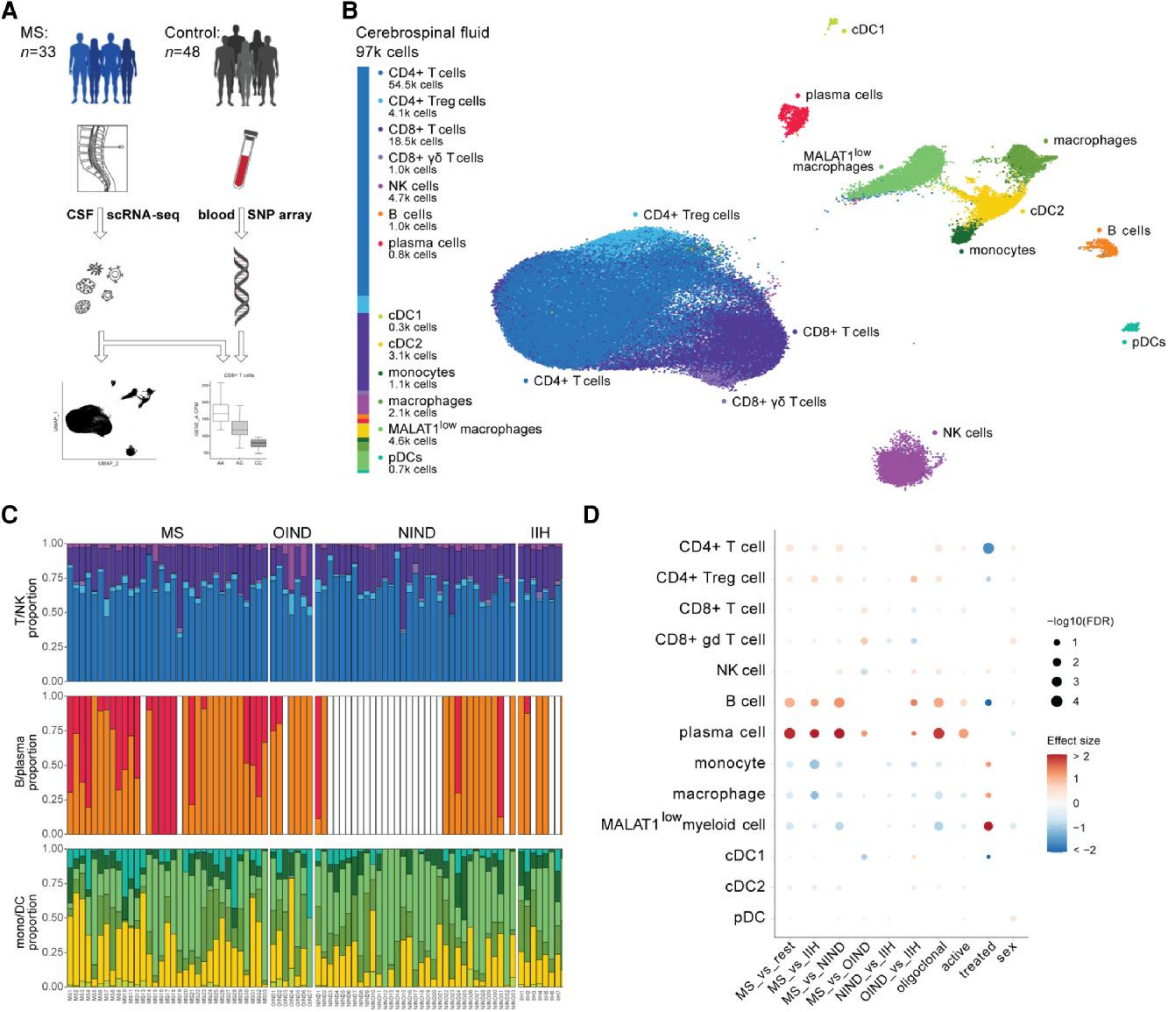
**Study overview and characterization of cellular architecture of CSF.** (**A**) Illustration of the experimental design and sampling scheme, in which droplet-based single cell sequencing was performed in 33 multiple sclerosis patents and 48 controls using the 10x Chromium Single Cell 3′ Solution V2. (**B**) Uniform manifold approximation and projection (UMAP) representation of single-cell transcriptomes of 96 732 CSF cells collected from all 81 donors, with the colours denoting the major cell types defined by iterative clustering and the expression of canonical marker genes. (**C**) Cell type proportions in three groups of cells across samples [T/natural killer (NK) at the *top*, B/plasma in the *middle* and monocytes/dendritic cells (DCs) at the *bottom*], ordered by sample group [multiple sclerosis (MS), other inflammatory neurological disease (OIND), non-inflammatory neurological disease (NIND) and ideopathic intracranial hypertension (IIH)]. Vertical bars correspond to individual samples. White indicates missing information (no cells of that type sampled in that individual). The colours correspond to the major cell types identified in **C**. (**D**) Differential cell abundance in pairwise comparisons between: MS versus non-MS; MS versus IIH; MS versus NIND; MS versus OIND; NIND versus IIH; OIND versus IIH; oligoclonal positive MS versus oligoclonal negative MS; active MS versus inactive MS; Tysabri-treated MS versus non-treated MS; female MS versus male MS patients. Dot colour denotes effect size estimates and dot size denotes the significance of the respective comparison. DC = dendritic cell; cDC1 = myeloid/conventional DC1; cDC2 = myeloid/conventional DC2; pDC = plasmacytoid DC; SNP = single nucleotide polymorphism.

We undertook single-cell transcriptome profiling of CSF cells from 33 multiple sclerosis patients and 48 with other neurological conditions using the 10x Genomics 3′ v2 platform (Fig. 1A and Supplementary Table 1). Following quality control, doublet removal and computational adjustment of ambient RNA (see the ‘Materials and methods’ section), a total of 96 732 cells were available for downstream analysis. On average, we captured 1194 quality control (qc)-passing cells per patient (range 21–10 491 cells per patient; Supplementary Table 2); 48 675 cells from patients with multiple sclerosis, 18 524 from patients with OIND, 13 847 from patients with NIND and 15 686 from patients with IIH.

Through dimensionality reduction, iterative unsupervised clustering and inspection of canonical marker gene expression, we annotated cells into 13 major immune populations which were used for all downstream analysis (Fig. 1B; see the ‘Materials and methods’ section). Most of our multiple sclerosis patients (*n* = 31) were treatment-naïve, the other two patients receiving treatment with Tysabri, a monoclonal antibody against α4-integrin which reduces the migration of leucocytes through the blood–brain barrier.^32^ This treatment is known to alter cell type composition in the CSF^7^; therefore, the Tysabri-treated patients were excluded from the cell type proportion analysis. Comparison of the immune cell type proportions between disease groups was concordant with previous findings,^3-7^ with multiple sclerosis patients having an overrepresentation of plasma cells, and an underrepresentation of monocytes and macrophages in comparison to patients with IIH and NIND (Fig. 1C and D). An increase in B cells and T regulatory cells was also seen in multiple sclerosis patients when compared to IIH and NIND but not OIND patients. Next, we assessed the relationship between cell type proportions within multiple sclerosis patients based on the clinical annotations of these samples (Fig. 1D). B and plasma cells were enriched in oligoclonal-positive multiple sclerosis patients and those with active disease. Whereas the Tysabri-treated patients showed a reduction of CD4+ T cell and B cell proportion when compared to treatment-naïve multiple sclerosis patients, correlating with previous findings of altered immune cell composition in the CSF of Tysabri-treated patients.^7^ A high proportion of macrophage-like cells characterized by very low expression of the long non-coding genes *MALAT1* and *NEAT1* was also observed in the Tysabri-treated multiple sclerosis patients (Fig. 1D). Published data would suggest that this population could be immunosuppressive as knockdown of *MALAT1* has been shown to reduce inflammatory injury following lung transplant through inhibiting the neutrophil infiltration and activation,^33^ while *NEAT1* knockdown was shown to lead to increased viral loads.^34^

Our higher resolution annotation identified many of the known low frequency cell subsets, including *AXL+* dendritic cells, *ACY3+* dendritic cells and *SPP1+* macrophages (Supplementary Figs 1 and 2), and further identified a less well defined rare population of CD8+ T cells expressing cytotoxic markers (*GZMA*, *GZMK*) and characterized by upregulation of the co-stimulatory marker *CD27*, upregulation of inhibitory receptors (*HAVCR2*, *TIGIT*) and downregulation of chemokines (*CCL4*, *CCL5*). This uncommon cell subtype was enriched in multiple sclerosis patients (Supplementary Fig. 3), however given the low frequency of these cells additional data will be required to confirm these initial findings. The patient specific high resolution cell type proportions are provided in Supplementary Table 2.

### Differential regulation of inflammatory and type I IFN gene expression programs in multiple sclerosis

**Figure 2:**
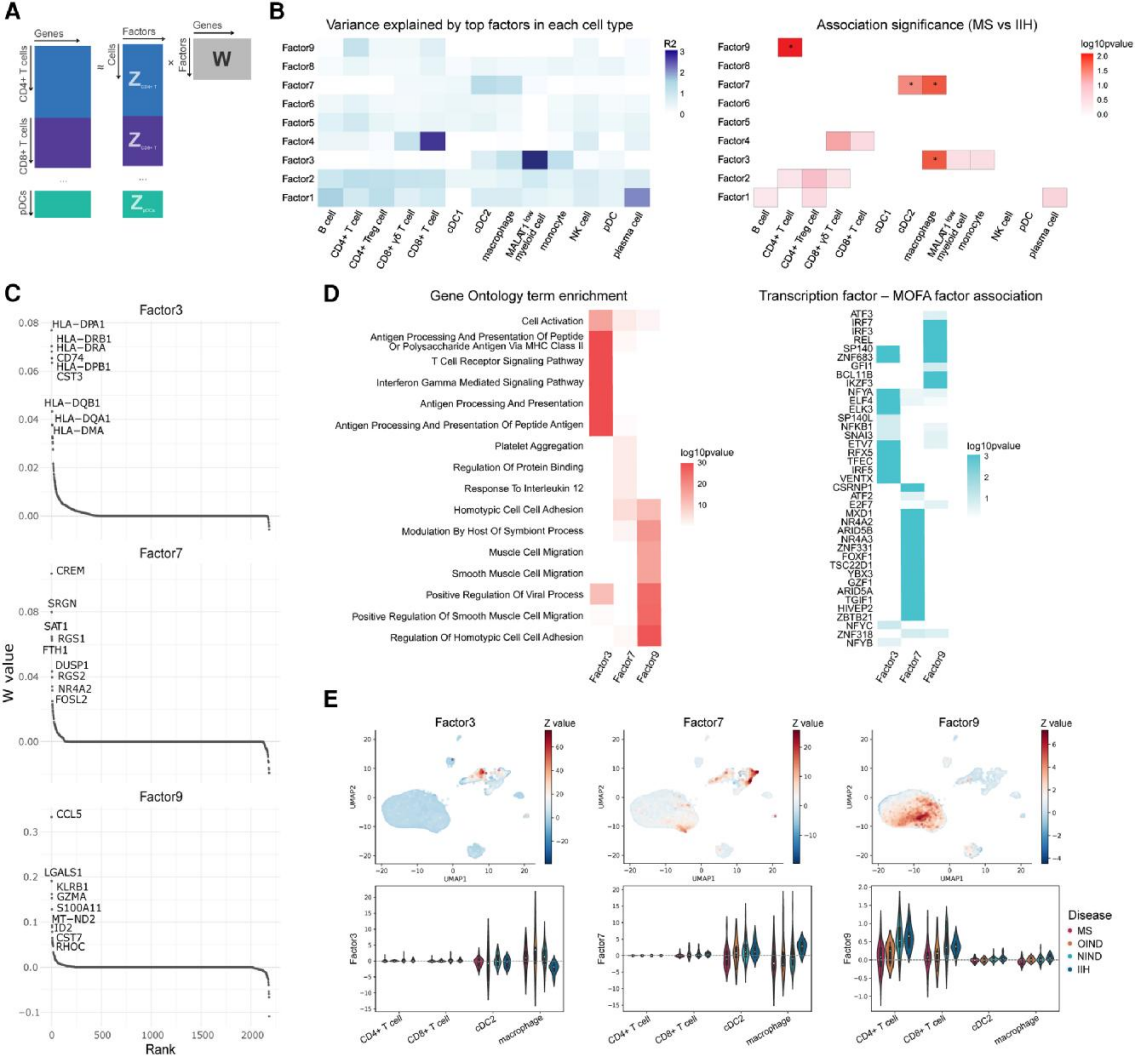
**Differential activity of gene expression programmes in multiple sclerosis versus controls.** (**A**) Illustration of the cell-type aware group-matrix factorization considered using multi-omics factor analysis (MOFA+). Expression profiles for different cell types are factorized into factors (Z) and weight matrices (W), respectively. (**B**) Gene expression variance explained by individual factors across cell types (R^2^) (*left*) and association with disease groups [multiple sclerosis versus ideopathic intracranial hypertension (IIH); linear model] (*right*). This analysis identifies factor 3 as primarily capturing disease-associated variation in macrophages, factor 7 as explaining variation in cDC2 and macrophages and factor 9 as explaining variation in CD4+ T cells. (**C**) Top gene loadings of the three disease-associated factors. (**D**) *Left*: Gene set enrichment based on factor loadings for the corresponding factors as in **C**. *Right*: A complementary strategy for factor annotations based on enrichments with known transcription factor targets. (**E**) Analysis of factor relevance across cells. *Top*: Factor activity across cells on the global uniform manifold approximation and projection (UMAP) representation coloured by the factor value weight; *bottom*: violin plots showing factor values averaged per donor show disease association for individual cell types. DC = dendritic cell; cDC1 = myeloid/conventional DC1; cDC2 = myeloid/conventional DC2; pDC = plasmacytoid DC; IIH = ideopathic intracranial hypertension; MHC = major histocompatibility complex; MS = multiple sclerosis; NK = natural killer cell; NIND = non-inflammatory neurological disease; OIND = other inflammatory neurological disease.

Next, we set out to characterize cell type-specific differences in function between multiple sclerosis cases and controls as reflected in gene expression. Given the limited power of conventional cell type-specific differential expression analysis of individuals we applied MOFA+^20,35^ to increase statistical power. This approach identifies sets of co-regulated genes (factors) that contribute substantially to gene expression variation within different cell types (Fig. 2A andSupplementary Table 3; see the ‘Materials and methods’ section). MOFA+ identified nine major factors, of which we focused on the 14 factor-cell type pairs that explained at least 1% of the variance in expression, and tested these for association with multiple sclerosis using the IIH patients as controls (see the ‘Materials and methods’ section). At a FDR of <10%, using linear regression and permutation-based statistics, we identified three multiple sclerosis-associated factors: factor 9 in CD4+ T cells, factor 3 in macrophages and factor 7 in macrophages and dendritic cells (Fig. 2B), the top-weighted genes for each factor are shown in Fig. 2C. To annotate these factors, we considered two complementary strategies. First, we performed a gene set enrichment analysis (GSEA) for each factor, weighting genes by the absolute value of their factor weights. Second, we searched for transcription factors (TFs) with regulons significantly enriched for genes with high factor weights (Fig. 2D), hypothesizing that these TFs might drive the co-regulated expression.^36^

The most statistically significant association with disease was observed for factor 9 in CD4+ T cells (*P* = 0.007; permutation test at the donor level), where patients with multiple sclerosis had reduced expression compared to IIH (Fig. 2E). The genes with the highest weights for this factor, including *CCL5*, *LGALS1*, *S100A11*, *GZMA* and *GZMK* (Fig. 2C), are characteristic of tissue-resident cytotoxic T effector memory cells^6,37^ and overlap with a previously described CD4+ T effector memory population re-expressing CD45RA (T_EMRA_).^38^ For this factor, GSEA shows significant enrichment for genes involved in the regulation of homotypic cell-cell adhesion (GO: 0034110; Fig. 2D). In T cells, homotypic cell interactions lead to the release of IL4, IL-10 and TGFβ and the generation of suppressor T cells, providing a negative feedback mechanism that controls immune responses.^39^ Several TFs involved in attenuating inflammatory and anti-viral responses have regulons overlapping with the genes showing the highest absolute weightings for factor 9 (Fig. 2D), including *BCL11B*,^40,41^*REL*,^42^*IKZF3*^43^ and *IRF3*/*IRF7*.^44^

The genes with the highest absolute value of weighing for factor 7 are characteristic of anti-inflammatory M2 macrophages, including *CREM*,^45^*FTH1*^46^ and NR4A2,^47^ and show reduced expression in multiple sclerosis. For factor 3, the genes with the highest absolute value weightings were upregulated in multiple sclerosis in macrophages and include a range of HLA genes and showed GSEA for antigen processing and presentation of peptide or polysaccharide antigen via MHC class II (GO:0002504) (Fig. 2D) as well as significant overlap with the regulon of *VENTX* which is involved in macrophage differentiation and pro-inflammatory activity.^48^ The regulons from TFs *IRF5* and *ETV7*, which are involved in type I IFN response regulation and are associated with M1 macrophage polarization,^49,50^ showed significant overlap for factor 3.

### Alignment of viral genomes is not enriched in patients with multiple sclerosis

**Figure 3:**
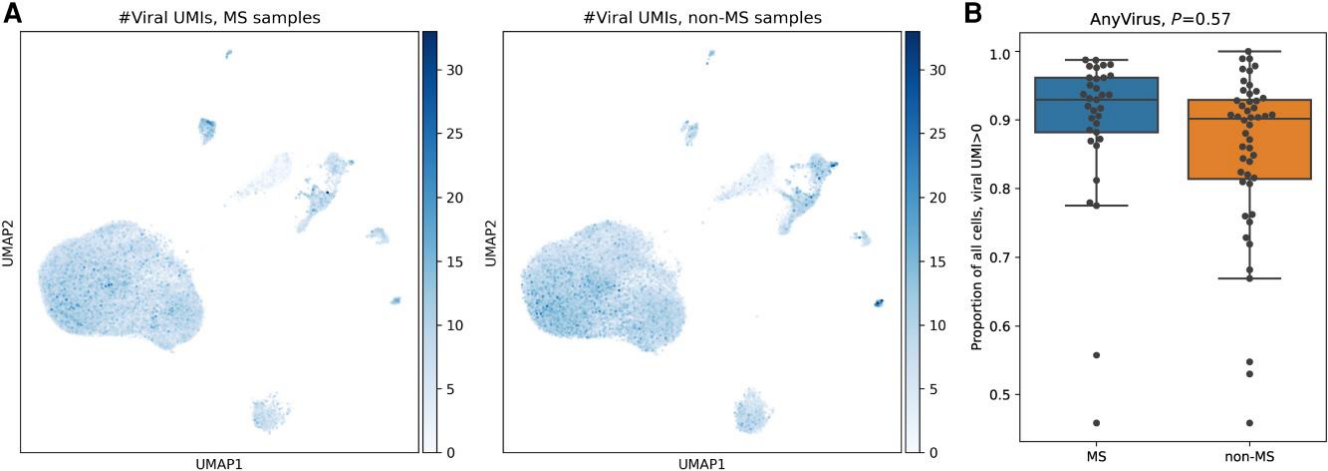
**Viral transcript expression in the CSF.** (**A**) Uniform manifold approximation and projection (UMAP) plots colour-coded for the total unique molecular identifier (UMI) counts per cell for sequence reads mapped to any of the 833 viral sequences analysed. *Left*: UMAP plot for the 48 675 cells from multiple sclerosis patients; *right*: UMAP plot for the 48 057 cells from all non-multiple sclerosis patients. (**B**) Proportion of cells that contain at least one viral UMI in multiple sclerosis patients versus controls, no statistically significant difference was found between the groups (*P* > 0.05). MS = multiple sclerosis.

Viruses, particularly the Epstein–Barr virus (EBV), have long been implicated in the pathogenesis of multiple sclerosis,^51^ and in comparison to healthy individuals the CSF from patients with multiple sclerosis more frequently contains polyspecific immunoglobulins directed against a range of common viral agents; the so-called Measles Rubella Zoster reaction.^52^ It therefore seemed logical to examine whether there was evidence of viral genomes in our CSF samples. To do this, we aligned the scRNA-seq reads that were either unmapped to the human transcriptome or were within unannotated regions in our data to 833 viral genomes and calculated the virus-specific UMI count per cell. We detected alignment (defined by at least one UMI detected across any cell type) for 78 of these viral genomes (Fig. 3A). The proportion of cells containing at least one viral UMI of any viral sequence showed no statistically significant difference between participants with multiple sclerosis compared to those without multiple sclerosis (Fig. 3B). Focusing on the specific viral genomes that were prevalent in more than 5% of either the multiple sclerosis or non-multiple sclerosis cohort (Supplementary Table 4 and Supplementary Fig. 4), the vast majority of these 65 well-represented viruses belong to the human endogenous retrovirus (HERV) family, transposable elements derived from retroviral integration into the human genome.^53^ Sequences of retroviral origin are considered to constitute up to 8% of the human genome.^54^ Despite being viewed as of little functional importance, HERVs are widely expressed in normal tissue particularly in the brain and testis^55^ and implicated in diseases including multiple sclerosis.^56^ While we observed an increased prevalence of a few HERVs in multiple sclerosis patients (Supplementary Table 4), this increase was not statistically significant (FDR > 10%). Outside of HERVs, the only other viruses that we identified in more than 5% of either the multiple sclerosis or non-multiple sclerosis cohort were the human herpesvirus 1, 2, 6A and 6B, human papillomavirus 71 and Abelson murine leukemia virus. While these were all more prevalent in multiple sclerosis, again this increase was not statistically significant (Supplementary Fig. 4 and Supplementary Table 4).

### Expression quantitative trait loci mapping in CSF cell types

To determine the impact of genetic variation on gene expression in the annotated cell types, we also completed array-based genome-wide genotyping of all individuals included in the study. Following imputation using the Haplotype Reference Consortium^10^ and quality control, we retained 9 149 816 common germline variants (minor allele frequency > 10% and MACH R2: 0.6; see the ‘Materials and methods’ section; Supplementary Fig. 5). We completed a genome-wide survey of eQTLs to uncover if there were any novel findings within cells isolated from the CSF. To do this, we applied the single-cell eQTL calling workflow proposed by Cuomo *et al*.^27^ Briefly, this approach maps *cis*-eQTLs in individual cell types by aggregating expression profiles at a donor level (considering between 6594 and 12 305 protein-coding genes per cell type and variants within 100 kb of each considered gene; see the ‘Materials and methods’ section). In CD4+ and CD8+ T cells, the most abundant cell types, we found 437 and 252 genes with a *cis*-eQTL, respectively, while for B cells, an uncommon cell type in the CSF, we only identified 25 such effects (Supplementary Tables 5 and 6). As the power for eQTL discovery is affected by the number of cells and individuals assayed, these results were not unexpected.

**Figure 4:**
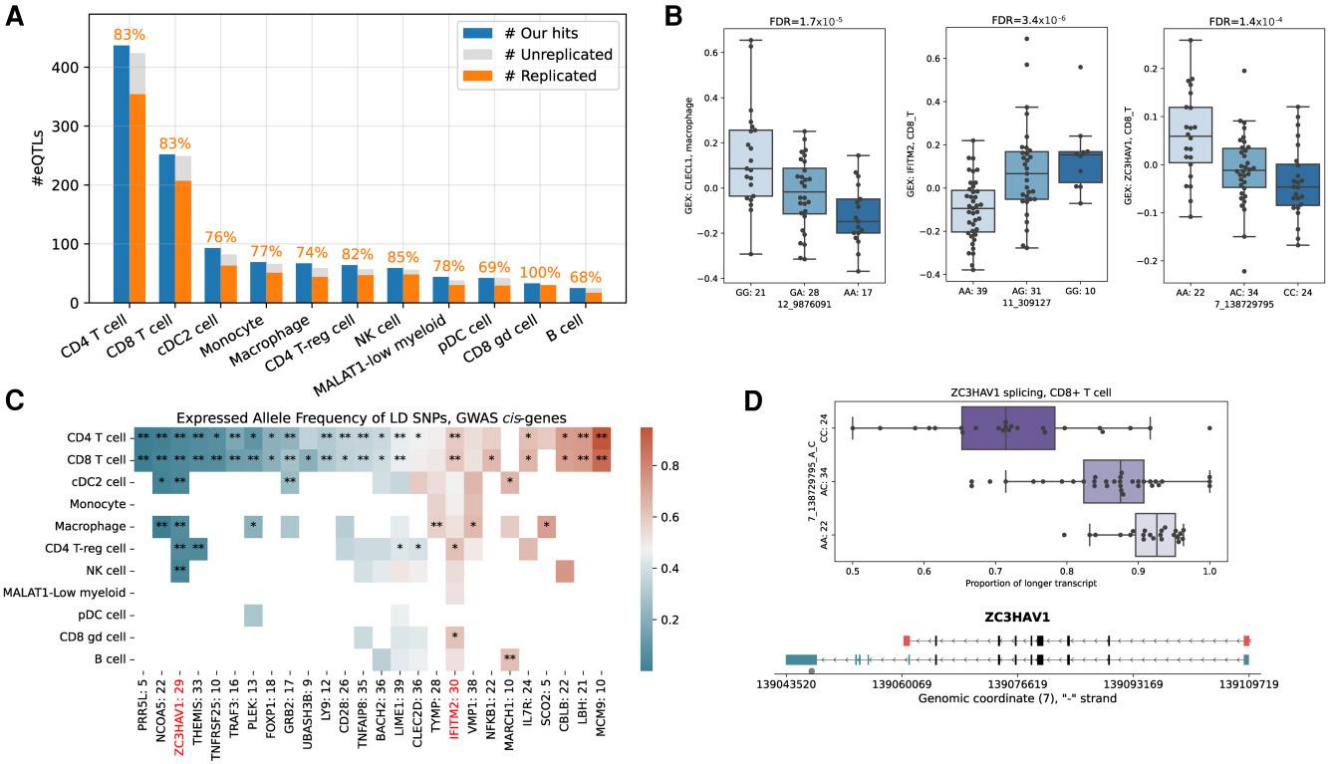
**Genetic effects on gene expression and co-localization with multiple sclerosis variants.** (**A**) Number of genes with a *cis*-expression quantitative trait loci (eQTL) detected across 11 cell types (blue bar). For genes that could be probed for replication (see the ‘Materials and methods’ section), the number of eQTLs that were consistent (orange bar) or discordant (grey bar) with previous studies in either peripheral blood mononuclear cells^58^ or cell type specific single cell data^57^ is indicated. Values above the bar denote the replication rates. (**B**) Scatter plot of gene expression, stratified for eQTL variant status, for the three genes with a cell-type-specific *cis*-eQTL fine mapped to established multiple sclerosis susceptibility variants. (**C**) Heatmap indicating allele-specific expression of the minor allele for the 26 single nucleotide polymorphisms (SNPs) in high linkage disequilibrium (LD) with multiple sclerosis-associated variants in different cell types with red indicating higher expression of the minor allele. The number of donors with heterozygous genotypes is indicated next to the gene name. White indicates a missing value where the gene was expressed <2 heterozygous donors. Single asterisk = false discovery rate (FDR) < 0.01; two asterisks = FDR < 0.0001 (Wald test, beta-binomial regression). (**D**) *Top*: Expression scatter plot showing the proportion of the long and short *ZC3HAV1* transcripts according to rs10271373 genotype; *bottom*: graphical representation of the two main *ZC3HAV1* transcripts. DC = dendritic cell; cDC2 = myeloid/conventional DC2; pDC = plasmacytoid DC; GWAS = genome-wide association study; NK = natural killer cell.

To assess the validity of our CSF eQTL signals, we sought to replicate these associations in relevant published cell-type specific single-cell^57^ and bulk RNA-peripheral blood mononuclear cell^58^ expression studies. As the cell types, genes and variants tested were not the same across these studies, we established the rate at which our CSF eQTLs were reported as eQTLs in either of these studies,^57,58^ with eQTLs considered to be confirmed if there was the same direction of effect at nominal significance (*P* < 0.05). Overall, 68%–100% of the CSF eQTLs we identified were confirmed in either of the published datasets (Fig. 4A andSupplementary Table 6). The highest replication rates were seen in T and natural killer cells (>82%), whereas cells derived from the myeloid lineage had a slightly lower replication rate (ranging from 69% to 79%), with the lowest replication seen for eQTLs in B cells (68%). Next, we looked for concordance of eQTL signals between CSF cell types (see the ‘Materials and methods’ section) and found that confirmation rates between cell types found in the CSF within our study ranged from 52% in B cells to 84% in CD4+ T regulatory cells (Supplementary Table 6). Given that eQTL effects are known to be influenced by the cell state and environment, we would not have expected complete concordance between blood and CSF or across the different cell types. In summary, the replication rate gives confidence that the eQTL signals described are genuine, despite the moderate sample size of our study.

Next, we used fine-mapping to investigate the relationship between the eQTL identified and the 200 multiple sclerosis susceptibility variants identified from GWAS.^1^ Using SusieR,^30^ we identified credible sets of causal variants for 416 of the 749 *cis*-eQTLs (Supplementary Table 7; see the ‘Materials and methods’ section); these sets contained a median of 21 variants per eQTL (ranging from 1 to 336). Of these 416 fine-mapped *cis*-eQTLs, 17 mapped within 100 kb of a known multiple sclerosis risk factor. In three instances, the credible set for the eQTL included the known multiple sclerosis susceptibility variants: rs3764021 for the expression of *CLECL1* in macrophages; rs1059091 for the expression of *IFITM2* in CD8+ T cells; and rs10271373 for the expression of *ZC3HAV1* in CD8+ T cells (Fig. 4B). We further investigated whether there was correlation of the fine-mapped eQTLs with genetic variants that have recently been described as associated with multiple sclerosis progression.^59^ Of the 12 progression variants, none mapped to within 100 kb of a fine-mapped *cis*-eQTL. This is perhaps not surprising, as enrichment analysis suggests the variants associated with progression are enriched in or near genes highly expressed in the brain which are not well captured in cells of the CSF.

### Allele-specific expression is consistent with expression quantitative trait loci mapping

To complement the eQTL analysis and increase our power to identify further *cis*-eQTL effects that are correlated with multiple sclerosis susceptibility or progression we completed an ASE analysis. No informative SNPs mapped close to the variants associated with multiple sclerosis progression [with linkage disequilibrium (LD) R^2^ > 0.15]; however, informative SNPs were available in 254 of the genes mapping close to multiple sclerosis susceptibility variants (with LD R^2^ > 0.15). Statistically significant evidence of ASE was seen for 40 of these genes in at least one cell type (Wald test in beta-binomial regression; FDR < 0.01; Supplementary Table 8), including 26 genes that have ASE in at least one cell type other than CD4+ T cells (Fig. 4C). Interestingly, in keeping with the eQTL analysis, we found evidence of ASE for both *ZC3HAV1* and *IFITM2*. In both genes the multiple sclerosis associated SNPs are located within transcribed regions of the gene [3′ untranslated region (UTR) and missense variant, respectively] and so were directly assessable for ASE. For *ZC3HAV1*, while the most statistically significant effect was seen in CD8+ T cells (correlating with the eQTL analysis), ASE was detected across most cell types with the same direction of effect. Meanwhile the ASE for rs1059091 in *IFITM2* was strongest in CD8+ T cells but also evident in CD4+ T cells alone, suggesting that the effects observed may be specific to these cell types. This aligns with publicly available data in peripheral blood mononuclear cells which demonstrate this SNP as an eQTL for *IFITM2* in CD8+ naïve T cells.^60^

Interestingly, we noted that the pattern of extensive ASE within *ZC3HAV1* was specifically localized to the 3′UTR of the longer *ZC3HAV1* transcript (ENST00000464606.5), which contains the multiple sclerosis-associated SNP rs10271373 as well as seven other SNPs in high linkage disequilibrium which all showed ASE. Alternative polyadenylation of *ZC3HAV1* results in multiple isoforms differing at the 3′ end of the gene. To further characterize whether the observed *ZC3HAV1* eQTL was a splice variant we used the BRIE software^31^ to quantify the proportion of the captured isoforms of *ZC3HAV1* (ENST00000464606.5/ENST00000680309.1 versus ENST00000471652.1). This analysis revealed that the eQTL influence of rs10271373 was mediated almost exclusively through an effect on the longer transcripts (ENST00000464606.5/ENST00000680309.1), which includes three additional exons (exon 10–12) and an alternate 3′UTR (Fig. 4D). The proportion of the longer transcript is significantly decreased from 91.3% in homozygotes for the multiple sclerosis risk allele to 73.8% in homozygotes for the protective allele (*P* < 1 ×10^−16^, Wald test in beta-binomial regression). The transcript usage does not differ between multiple sclerosis and non-multiple sclerosis samples in any cell type (*P* = 0.42).

## Discussion

Here we report the results from our comprehensive single-cell resolution analysis of CSF from patients with multiple sclerosis and other neurological diseases. Our analysis confirms the well-established cellular composition of CSF in patients with multiple sclerosis and highlights inflammatory responses altered in multiple sclerosis. We further describe the first single-cell eQTL analysis of CSF and identify two eQTLs in CD8+ T cells correlated with multiple sclerosis susceptibility, both in genes related to controlling viral responses—*ZC3HAV1* and *IFITM2*.

Our eQTL analysis indicates that the multiple sclerosis susceptibility variant rs10271373 influences the expression of *ZC3HAV1* in CD8+ T cells, a gene with four isoforms,^61^ two of which, ZAP-L and ZAP-S,^62^ are suggested to be important in resistance to particular viruses.^62-65^ This eQTL effect appears to be driven by an underlying splicing QTL, with increased usage of the ZAP-L isoform in individuals that carry the multiple sclerosis risk allele (rs10271373_A). This longer isoform has an extended C-terminal region which contains a poly (ADP ribose) polymerase (PARP)-like domain^62^ and contains a CaaX prenylation motif. Farnesyl modification of the cysteine residue of this motif enhances the antiviral activity through altering the subcellular location of ZAP-L to the endocytic and lysosomal compartments for viral interaction.^66^ Interestingly, this isoform is known to be under positive selection^62^ and contains EBV miRNA binding sites within its 3′UTR.^67^ We further identified a second eQTL that fine-mapped to a multiple sclerosis susceptibility variant (rs1059091) for another antiviral gene, *IFITM2*, where reduced expression of the gene in CD8+ T cells was observed in individuals carrying the risk allele (rs1059091_A), supporting a previous finding that observed this eQTL in CD8+ naïve T cells only,^60^ suggesting this eQTL is likely to be cell-type specific. *IFITM2* is believed to inhibit viral entry, likely through effects on the endocytic pathway^68^ and has also been implicated in Th2 T cell differentiation.^69^ Given the location of *IFITM2* within the IFITM cluster on chromosome 11, along with transcript-specific genotypic effects of rs1059091 on *IFITM2* expression in response to particular viral strains,^70^ further investigation is warranted to investigate this complex regulatory region.

Given the likely importance of viral infection in the pathogenesis of multiple sclerosis,^51^ especially the role of EBV,^71^ it seemed logical to check our samples for the presence of viruses. While we did not identify an enrichment of any one virus in the CSF of patients with multiple sclerosis, patients with multiple sclerosis did have an increased proportion of a rare CD8+ T cell population where the top defining markers, including the inhibitory receptors *HAVCR2* and *TIGIT*, overlap previously described viral specific exhausted-like CD8+ cells,^72,73^ which are known to arise following chronic antigenic stimulation.^74^ The expression of *HAVCR2* and *TIGIT* in CD8+ T cells however is also present on several other CD8+ T cell phenotypes, including activated cells,^75^ and tissue resident memory cells.^76^ Given that exhausted T cells^77^ and CD8+ tissue resident memory cells^7,8,78^ have all been implicated in multiple sclerosis, careful phenotyping of this cell population at the epigenetic and protein level will be essential to establish the function of these cells and in turn the mechanism by which they may be of relevance in the disease process.

Using a MOFA approach, we identified several factors showing altered expression in multiple sclerosis. The most statistically significant being the decreased activity of genes controlling anti-inflammatory and anti-viral type I IFN responses in the T cells. The genes with the highest MOFA weighting for this factor also overlap with those characterizing tissue-resident cytotoxic T cell effector memory^6,37^ or T_EMRA_ cells.^38^ This finding is consistent with previous work, emphasizing the importance in multiple sclerosis of specific T helper cell subsets,^79^ alongside the protective effects of type I IFNs in CNS autoimmunity and the known benefit of using the type I IFN—IFNβ—as a treatment in multiple sclerosis.^80^ Whether our observation that these genes show reduced expression in multiple sclerosis reflects a depletion of a specific cell subset or rather reflects a shift in function across a wider range of CD4+ T cells remains to be established. The limited ability to resolve cell subtypes in unstimulated CD4+ T single-cell data is well established.^81^ While reference sets have proven useful in partially overcoming this issue,^82^ development of disease-relevant reference sets will be required to allow meaningful cross-study comparisons of disease-specific effects within T cell subtypes.

As well as changes in T cells we also identified two disease-relevant MOFA factors in the macrophage population, one related to anti-inflammatory M2 polarization and the other related to antigen presentation. Macrophages have been shown to be of importance in the mouse model of multiple sclerosis—experimental autoimmune encephalomyelitis^83^—human multiple sclerosis pathology,^84^ disease progression,^85^ remyelination^86^ and in the neuro-inflammation present in many degenerative neurological diseases.^87^ Our MOFA data indicate that in multiple sclerosis there is reduced polarization towards the M2 phenotype and increased expression of the machinery of antigen presentation, likely indicating that the MOFA has identified factors indicative of an altered phenotypic cell state in multiple sclerosis.

Given the extreme functional plasticity of immune cells in response to environmental stimuli, it is difficult to disentangle whether the phenotypes we have captured represent the cause or the consequence of disease. However, our single cell analysis has identified genes and pathways critical in both adaptive and innate immune cells, emphasizing the importance of both arms of the immune system in multiple sclerosis. Furthermore, our finding that the multiple sclerosis susceptibility variants rs10271373 and rs1059091 are eQTLs for antiviral genes in CSF CD8+ T cells supports the hypothesis that dysregulation in viral control mechanisms is involved in the development of multiple sclerosis.

## Supplementary Material

awad404_Supplementary_Data

## Data Availability

These data have been deposited in the European Genome-phenome Archive (EGA), which is hosted by the EBI and the CRG, under accession number EGAS00001007478. For reproducibility, the data preprocessing scripts and analysis notebooks can be found at https://github.com/huangyh09/MSclerosisSrc. For the eQTL analysis, we used the standard pipeline at: https://github.com/single-cell-genetics/limix_qtl.
