# Supplementary material for: Expression profiling of cerebrospinal fluid identifies dysregulated antiviral mechanisms in multiple sclerosis
Source: Brain. 2023 Dec 1;147(2):554–65. doi: 10.1093/brain/awad404 (PMC10834244; doi:10.1093/brain/awad404)
Supplement: awad404_Supplementary_Data [file awad404_supplementary_data.zip › brain-2023-01551-File006.pdf]

Supplementary Figures

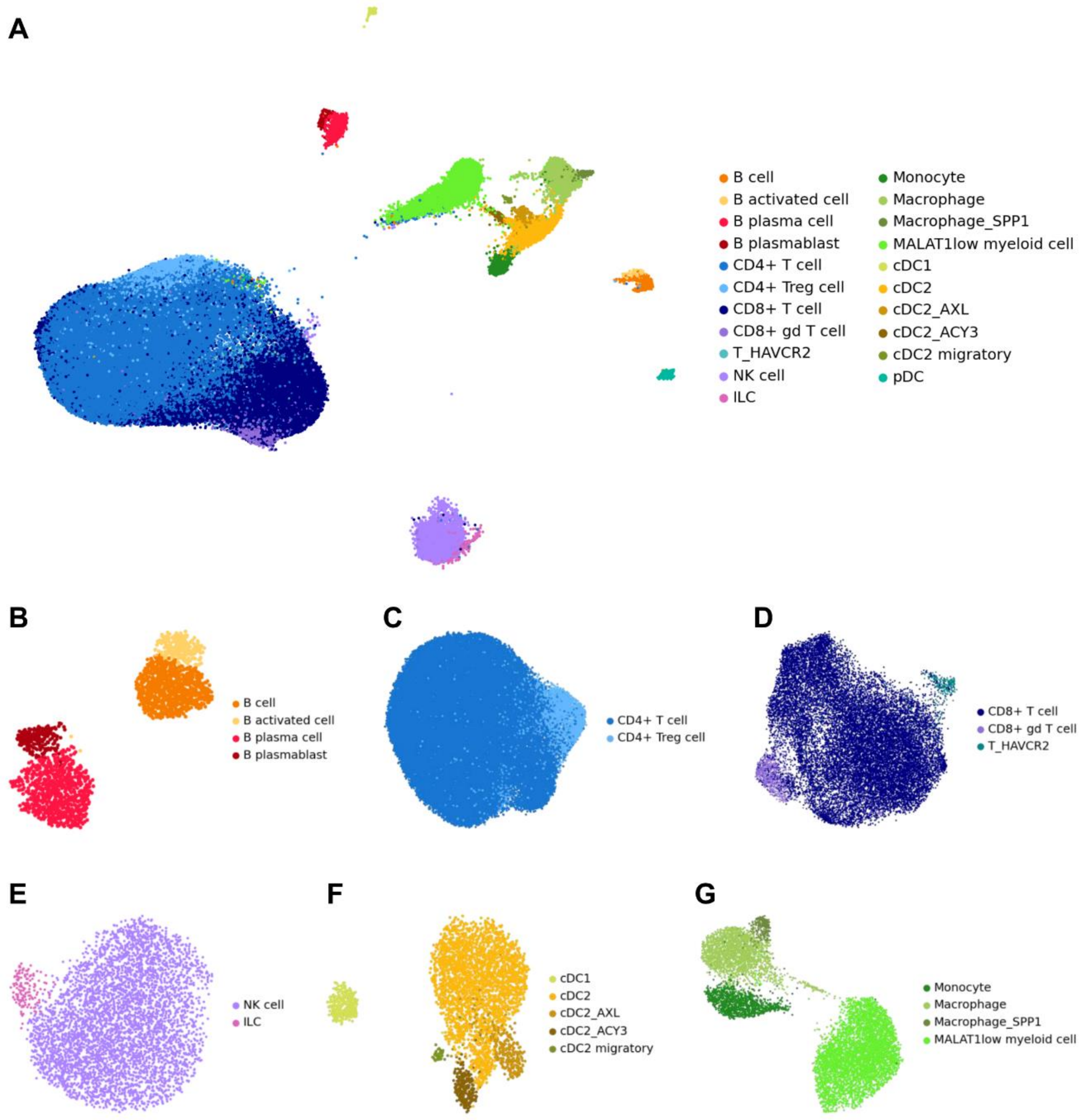

Supplementary Figure 1. Higher resolution annotation of the CSF cell types. (A) UMAP of all CSF cells coloured by higher resolution annotation. UMAP of B/plasma cell cluster **(B)**; CD4+ T cell cluster **(C)**; CD8+ T cell cluster **(D)**; NK cell cluster **(E)**; dendritic cells **(F)**; and Monocyte/Macrophages **(G)**

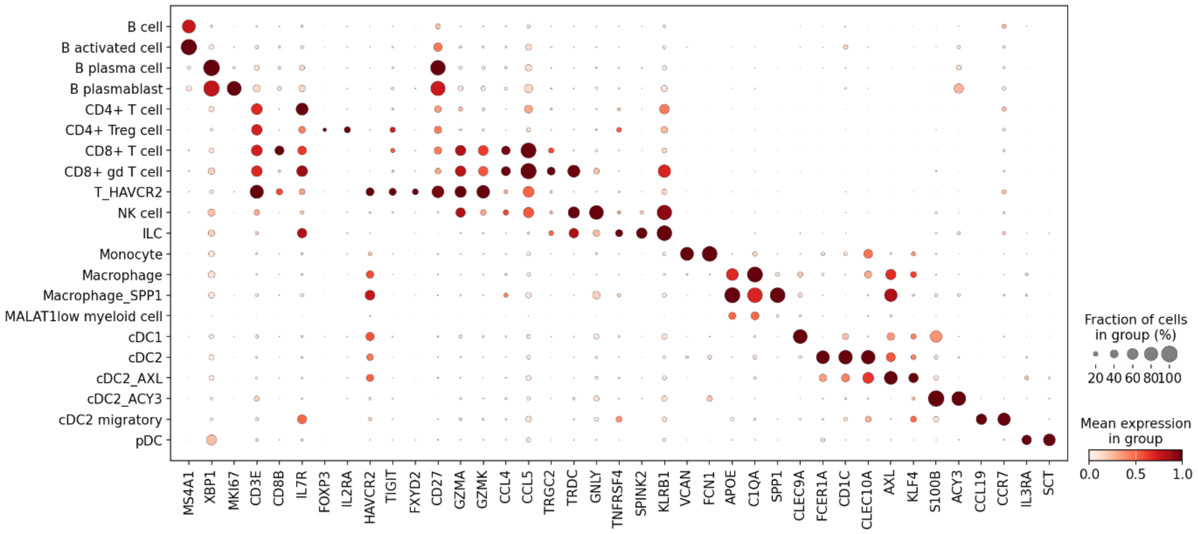

Supplementary Figure 2. Higher resolution cell-type defining gene expression. Dot plot showing the mean expression of cluster defining genes (x-axis) across our higher resolution cell type annotation (y-axis). The scaled mean gene expression in each cluster is indicated by the colour. The percent of cells expressing the gene is indicated by the dot size.

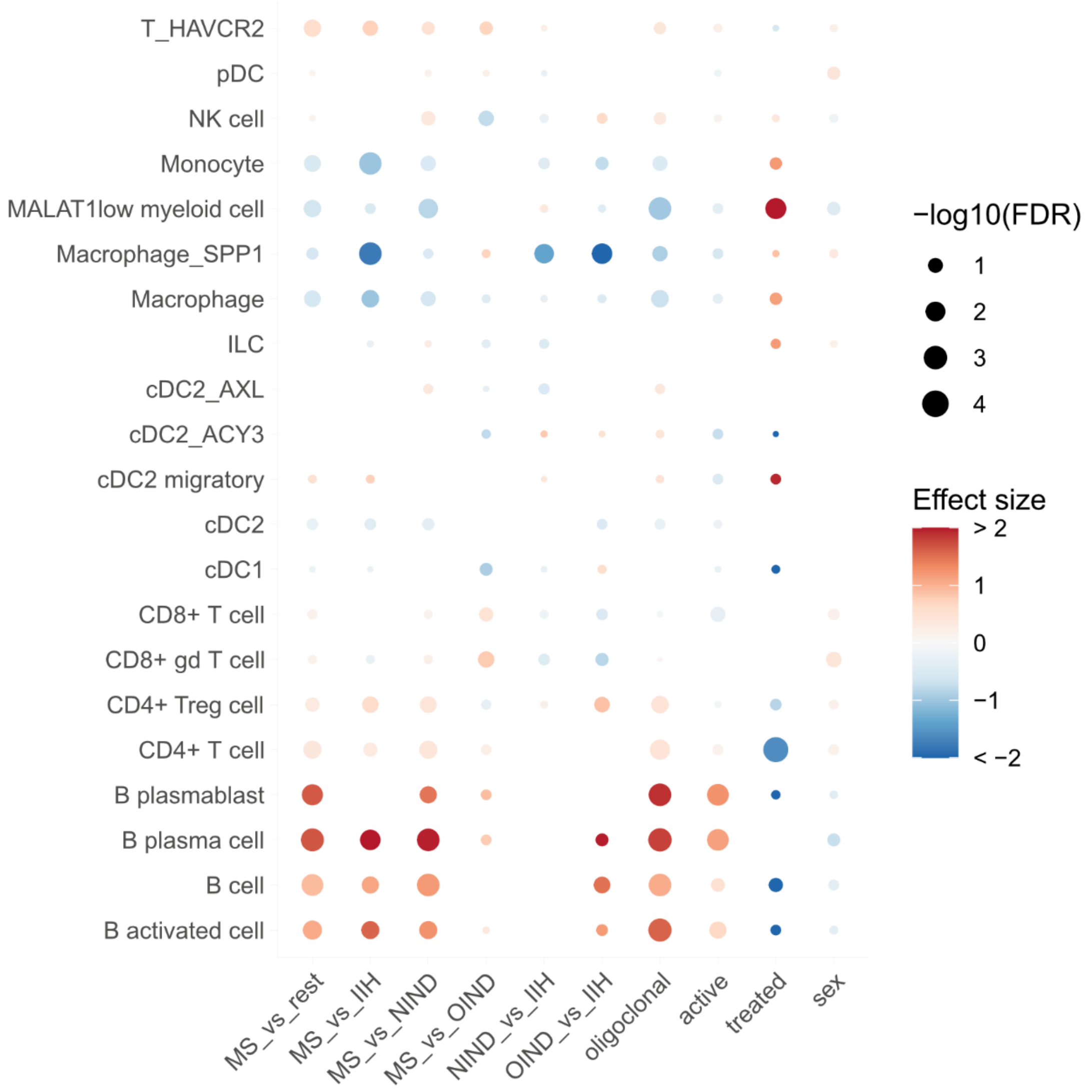

Supplementary Figure 3. Higher resolution annotation of cell-type proportions
Higher resolution differential cell abundance in pairwise comparisons between: MS vs non-MS; MS vs IIH; MS vs NIND; MS vs OIND; NIND vs IIH; OIND vs IIH; oligoclonal positive MS vs oligoclonal negative MS; active MS vs inactive MS; Tysabri treated MS vs non-treated MS; female MS vs male MS patients. Dot colour denotes effect size estimates and dot size denotes the significance of the respective comparison

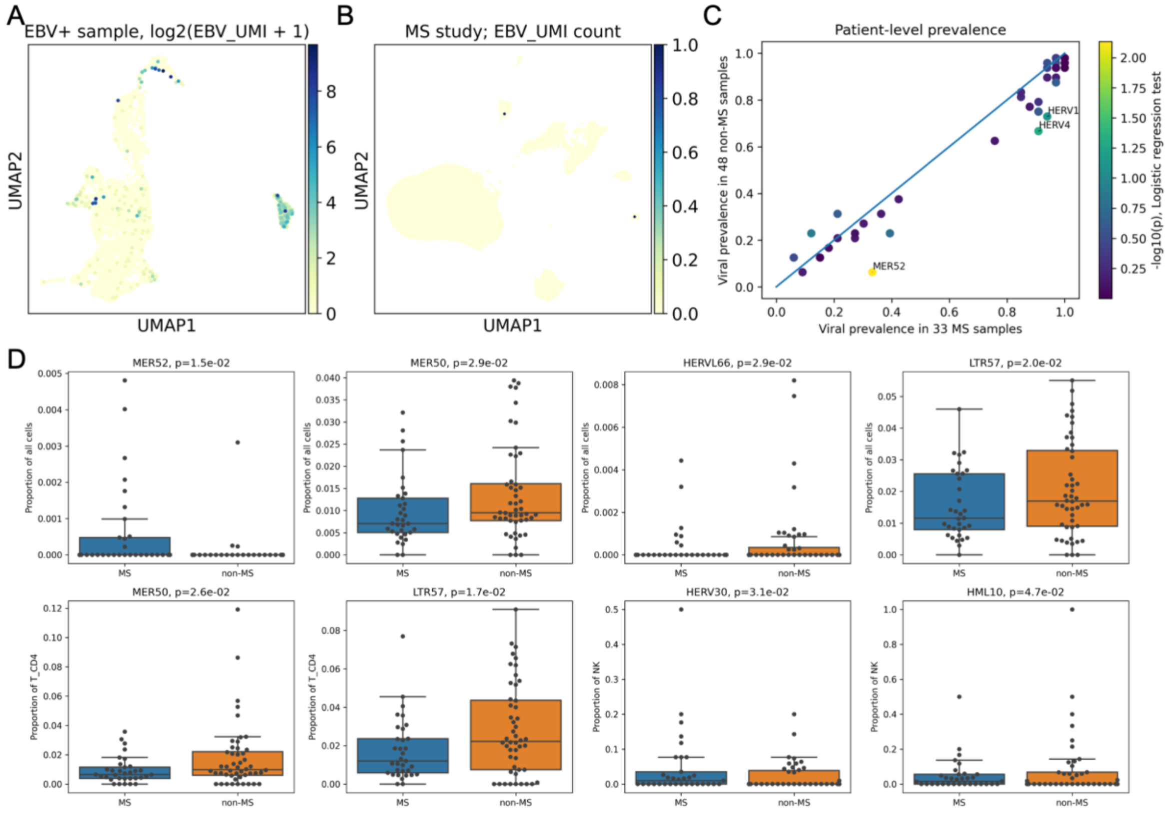

Supplementary Figure 4. Analysis of expressed viral transcripts across 833 viruses in the CSF.

In this pipeline, we extracted the reads that were not counted or mapped to the human transcriptome and remapped them to the sequences of 833 viruses (see Methods).

(A) UMAP plot for an EBV positive data set from a public database (GEO: GSE157243) with colour coded for log2(UMI + 1) value for reads mapped to Epstein-Barr virus (EBV). This serves as a positive control to assess the effectiveness of the pipeline.

(B) UMAP plot for the 96,732 cells from our 81 samples colour coded as in (A).

(C) The comparison of prevalence at patient level for 65 viruses that have >5% prevalence in either MS samples or non-MS samples. Three viruses were detected as having moderately different prevalence between MS and non-MS samples (p value < 0.1), but this difference was not statistically significant (all FDR>0.3).

(D) Proportion of cells positive for the 65 viruses in (C) between MS and non-MS samples in different cell types. The 8 virus-cell type pairs with p value <0.05 are shown, this number is not significant after correcting for multiple testing (all FDR>0.3).

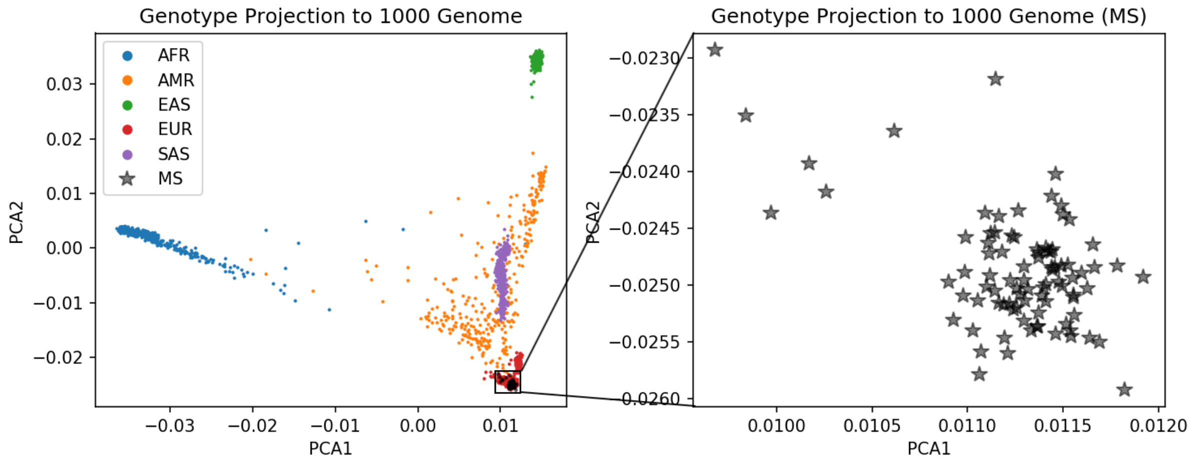

Supplementary Figure 5. PCA of the 81 CSF samples

Projection of our 81 samples (labelled as MS) onto the first two principal components (PC) of the genotypes from the 2,504 donors from the 1000 Genomes Project (ethnic groups denoted by colour). The PCs are calculated with plink on 56,700 genotyped SNPs with a minor allele frequency (MAF > 5%) and linkage disequilibrium (LD) pruned (50K window, 5 nt step, r^2<0.1) - the MHC region was excluded.
